# Isolation of Oxamyl-degrading Bacteria and Identification of *cehA* as a Novel Oxamyl Hydrolase Gene

**DOI:** 10.3389/fmicb.2016.00616

**Published:** 2016-04-29

**Authors:** Konstantina Rousidou, Eleni Chanika, Dafne Georgiadou, Eftychia Soueref, Demetra Katsarou, Panagiotis Kolovos, Spyridon Ntougias, Maria Tourna, Emmanuel A. Tzortzakakis, Dimitrios G. Karpouzas

**Affiliations:** ^1^Laboratory of Plant and Environmental Biotechnology, Department of Biochemistry and Biotechnology, University of ThessalyLarisa, Greece; ^2^Laboratory of Wastewater Management and Treatment Technologies, Department of Environmental Engineering, Democritus University of ThraceXanthi, Greece; ^3^Department of Viticulture, Vegetable Crops, Floriculture and Plant Protection, Institute of Olive Tree, Subtropical Crops and Viticulture, National Agricultural Research Foundation, Hellenic Agricultural Organization-DEMETERHeraklion, Greece

**Keywords:** oxamyl, microbial degradation, *Pseudomonas*, carbamate pesticides, *cehA* gene

## Abstract

Microbial degradation is the main process controlling the environmental dissipation of the nematicide oxamyl. Despite that, little is known regarding the microorganisms involved in its biotransformation. We report the isolation of four oxamyl-degrading bacterial strains from an agricultural soil exhibiting enhanced biodegradation of oxamyl. Multilocus sequence analysis (MLSA) assigned the isolated bacteria to different subgroups of the genus *Pseudomonas*. The isolated bacteria hydrolyzed oxamyl to oxamyl oxime, which was not further transformed, and utilized methylamine as a C and N source. This was further supported by the detection of methylamine dehydrogenase in three of the four isolates. All oxamyl-degrading strains carried a gene highly homologous to a carbamate-hydrolase gene *cehA* previously identified in carbaryl- and carbofuran-degrading strains. Transcription analysis verified its direct involvement in the hydrolysis of oxamyl. Selected isolates exhibited relaxed degrading specificity and transformed all carbamates tested including the oximino carbamates aldicarb and methomyl (structurally related to oxamyl) and the aryl-methyl carbamates carbofuran and carbaryl which share with oxamyl only the carbamate moiety.

## Introduction

Oxamyl [*N,N-dimethyl-2-methylcarbamoyloxyimino-2-(methylthio)acetamide*] is an oximino carbamate nematicide used for the control of nematodes in protected crops and potato cultivation. The recent withdrawal or restricted use of several nematicides like aldicarb, cadusafos, and fenamiphos, made oxamyl one of the leading synthetic non-fumigant nematicides in the global market. Oxamyl is a rather water soluble molecule (280 g L^-1^), which is only weakly sorbed by soil (*K*_om_ 21.9 ml g^-1^) and so is readily mobile for leaching to groundwater (Gerstl, 1984). It is characterized by high acute toxicity to mammals (LD50_oral_ rat = 2.5 mg kg^-1^) and aquatic organisms (EC50 48 h *Daphnia magna* = 0.319 mg L^-1^; European Food Safety Authority [EFSA], 2005).

Oxamyl is not considered a persistent chemical in soil. This has been illustrated by previous laboratory and field dissipation studies which reported half-life values of 8–60 days (Smelt et al., 1983; Ou and Rao, 1986) and 7–28 days (Ambrose et al., 2000), respectively. Microorganisms and pH are the two factors controlling the degradation of oxamyl in soils. It is well documented that the degradation of oxamyl is accelerated in neutral to alkaline soils (Bromilow et al., 1980; Smelt et al., 1983). This is the result of the vulnerability of oxamyl to abiotic hydrolysis (Harvey and Han, 1978) and the higher bacterial activities commonly observed under alkaline soil conditions (Smelt et al., 1987). In its extreme biodegradation could lead to the rapid dissipation of oxamyl in soils repeatedly treated with the nematicide and eventually to loss of its biological efficacy (Smelt et al., 1987). This phenomenon has been named enhanced microbial degradation and it has been attributed to the adaptation of a fraction of the soil microbial community to rapidly degrade oxamyl (Osborn et al., 2010a).

Soils exhibiting enhanced biodegradation of carbamates have been used as a source for the isolation of carbamate-degrading bacteria. The majority of earlier studies have focused on the isolation of bacteria degrading aryl methyl-carbamates like carbofuran and carbaryl (Karns et al., 1986; Bano and Musarrat, 2006; Yan et al., 2007). On the contrary, only a few bacterial strains able to degrade the oximino carbamates oxamyl, aldicarb, and methomyl have been isolated to date. Two methomyl-degrading bacteria were previously isolated from activated sludge (Xu et al., 2009) and water samples (Mohamed, 2009). Similar studies reported the isolation of two aldicarb-degrading bacterial strains which were identified as *Stenotrophomonas maltophilia (*Karayilanoglu et al., 2008) and *Methylosinus* sp. (Kok et al., 1999). Osborn et al. (2010b) first reported the isolation of 27 oxamyl-degrading bacteria identified as *Aminobacter* and *Mesorhizobium* spp. However, no details on the metabolic pathway and the genes involved in the degradation of oxamyl were provided.

Previous studies with carbamate-degrading bacteria have identified the genes involved in the hydrolysis of methyl carbamates. Tomasek and Karns (1989) first isolated a plasmid-encoded carbofuran-hydrolase gene *mcd* from an *Achromobacter* strain, which appeared to be widely spread in agricultural soils where carbofuran was applied (Parekh et al., 1996). Later studies by Hashimoto et al. (2002) reported the isolation of a plasmid-encoded esterase gene *cehA* from a *Rhizobium* strain, which was able to hydrolyze the methyl-carbamate carbaryl. Subsequent studies by Hashimoto et al. (2006) reported the isolation of an amidase-encoding gene *cahA* from an *Arthrobacter* strain, which was able to degrade carbaryl. No such information is available for oximino carbamates like oxamyl and the genes/enzymes involved in their microbial degradation are still unknown.

The main aims of this study were (i) to isolate and characterize oxamyl-degrading soil bacteria, (ii) to explore the microbial metabolic pathway of oxamyl, and (iii) to identify the genes involved in the hydrolysis of oxamyl.

## Materials and Methods

### Pesticides and Media

Analytical grade oxamyl (99.6%, Fluka, Switzerland), oxamyl oxime (100%, DuPont, USA) carbofuran, carbaryl (99%, ChemService, USA), methomyl, aldicarb, aldicarb sulfoxide, and sulfone (99.9%, Fluka, Switzerland), were used throughout this study. An aqueous solution of oxamyl (500 mg L^-1^ in sterile ddH_2_O) was used for the preparation of oxamyl-containing media. Bacteria were isolated using a selective mineral salts medium (MSM; Karpouzas et al., 2000), where oxamyl served as the sole C and N source. Mineral salts agar containing oxamyl (10 mg L^-1^) was prepared in a similar way to the MSM liquid media except that Bacto Agar (15 g L^-1^; LAB M, UK) was added. Nutrient Broth (NB) and Luria-Bertani broth (LB) were purchased from LAB M (UK) and they were prepared according to manufacturers’ instructions. Nutrient agar (NA) was prepared in a similar way to NB except that Bacto Agar (15 g L^-1^) was added.

### Soil Microbial Degradation of Oxamyl and Oxamyl Oxime

The degradation of oxamyl was investigated in a soil from a commercial banana plantation located in the area of Sitia, northeast Crete, Greece. The plantation had been treated with oxamyl twice a year for the last 5 years. Despite that, the farmer was facing severe infestation by the lesion nematode *Pratylenchus goodeyi* and the spiral nematode *Helicotylenchus multicinctus*. Approximately 400 g of sieved soil were divided into two sub-samples of 200 g. The first was sterilized in an autoclave at 121°C for 30 min. Both samples were then treated with an aqueous solution of oxamyl (500 mg L^-1^), aiming at a soil dose rate of 10 μg g^-1^. Water was then added to the treated soil samples to adjust their moisture content to 40% of their water holding capacity. The two soil samples were then separated into sub-samples of 20 g, which were placed in aerated plastic bags and incubated in the dark at 25°C. Immediately after pesticide application and at daily intervals thereafter, triplicates from each soil were analyzed for oxamyl and its metabolites via HPLC.

In a follow up experiment we investigated the involvement of microorganisms in the transformation of oxamyl oxime (hydrolysis product of oxamyl) in the same soil. Briefly, 120 g of sterilized and non-sterilized soil were treated with 8 mg kg^-1^ of oxamyl oxime and the samples were incubated in the dark at 25°C. Immediately after the addition of oxamyl oxime in soil and 10 days later, the levels of oxamyl oxime were determined by HPLC.

### Isolation of Oxamyl-degrading Bacteria

Oxamyl-degrading bacteria were isolated from soil via enrichment cultures (Karpouzas et al., 2000). At the point of 50% degradation of oxamyl in the fourth enrichment cycle, a 10-fold dilution series was prepared on triplicate MSM + oxamyl (50 μM) agar plates. After incubation at 25°C for 5 days, 50 single colonies were randomly selected and their degrading ability was assessed in MSM + oxamyl (50 μM) at 25°C by HPLC. Cultures showing more than 50% degradation of oxamyl during the first 7 days were considered positive and they were spread onto MSM + oxamyl (50 μM) and NA plates to test purity.

A follow up enrichment was established to isolate bacteria able to degrade the main hydrolysis product of oxamyl, oxamyl oxime. Triplicate flasks containing MSMN and MSM supplemented with 20 mg L^-1^ of oxamyl oxime (as a sole C or as a sole C and N source, respectively) were inoculated with 0.2 g of soil from the banana plantation which had been previously treated with oxamyl oxime. Duplicate non-inoculated controls were also included. The degradation of oxamyl oxime was followed by HPLC.

### Phylogenetic Classification of the Oxamyl-degrading Bacteria

Oxamyl-degrading isolates were grown in MSM + oxamyl (50 μM) agar plates for 4 days at 25°C. Bacterial cells were collected in 0.5 ml of sterile ddH_2_O and DNA was extracted using the Nucleospin Tissue kit (Macherey-Nagel, Germany). Multilocus sequence analysis (MLSA) of the housekeeping genes 16S rRNA, *rpoD*, and *gyrB* was used for the phylogenetic classification of the isolated bacteria. Details on the primers used for the amplification of the target genes are given in Supplementary Table S1. Amplification was carried out in 25-μl reactions containing 1U of DyNAzyme EXT^TM^ (Finnzymes), 0.2 μM of each primer, 1X buffer (DyNAzyme^TM^ EXT buffer), 1.5 mM of MgCl_2_, and 200 μM of each dNTPs. The PCR thermal cycling conditions for the amplification of the 16S rRNA gene were 95°C for 5 min, followed by 25 cycles of 95°C for 1 min, 55°C for 1 min, and 72°C for 2 min, with a final extension of 72°C for 10 min. PCR amplification of the *gyrB* and *rpoD* genes was performed as described by Mulet et al. (2009, 2010). The PCR products obtained were purified with the Nucleospin II PCR clean-up kit (Macherey-Nagel, Germany), cloned into plasmid vector pGEM – T easy (Promega, USA), and transformed into *Escherichia coli* (DH5a High Efficiency Competent Cells – Invitrogen, USA) following standard procedures (Sambrook et al., 1989). Plasmid DNA from at least three clones per isolate and gene were extracted using the NucleoSpin Plasmid kit (Macherey-Nagel, Germany) and sequenced (Cemia SA, Larissa, Greece).

For the phylogenetic analysis of the isolates, three alignments, one per housekeeping gene sequenced, were prepared by using clustalo^1^. Subsequently, the individual alignments (16S rRNA gene: 1346 nt; *gyrB* gene: 519 nt; *rpoD* gene: 648 nt) were merged in Mesquite ver. 2.75 (Maddison and Maddison, 2011) forming a unified matrix which was used to construct the concatenated phylogenetic tree. Evolutionary distances were calculated by the Jukes and Cantor (1969) and the phylogenetic trees were generated by the “neighbor-joining” method (Saitou and Nei, 1987). TREECON for Windows (Van de Peer and de Wachter, 1993) was used for tree construction from distance matrix. MLSA of the housekeeping genes *16S rRNA, gyrB*, and *rpoD* has been proposed as the most comprehensive method to establish phylogenetic relationships among the species in the genus *Pseudomonas* (Mulet et al., 2010; Gomila et al., 2015). Sequences of the 16S rRNA, *gyrB*, and *rpoD* genes of the isolates analyzed were deposited in the European Molecular Biology Laboratory (EMBL) database under the accession numbers FN600408 – FN600411 (16S rRNA gene), KT808454-KT808457 (*gyrB*), and KT808458-KT808461 (*rpoD*).

### Growth Kinetics and Oxamyl Degradation

In all studies bacterial inocula were prepared as described by Karpouzas and Walker (2000a). All cultures were incubated on a shaking platform at 150 rpm at 25°C. Triplicates containing 20 ml of MSM + oxamyl (50 μM) were inoculated with each one of the four isolates. Triplicates (10 ml) were amended with 0.2 ml of MSM without bacteria to serve as non-inoculated controls. The degradation of oxamyl and the formation of oxamyl metabolites were determined immediately after inoculation and at regular intervals thereafter. The initial inoculum density [3 × 10^6^ colony forming units (cfu) ml^-1^] and the subsequent growth of the oxamyl-degrading bacteria during degradation of oxamyl were determined by serial dilution plating in NA.

### Degradation of Other Pesticides by Oxamyl-degrading Bacteria

The ability of one of the isolated bacteria, strain OXA20, to degrade other carbamate pesticides was investigated. Aqueous solutions of carbofuran (200 mg L^-1^), methomyl (500 mg L^-1^), and aldicarb (500 mg L^-1^) were prepared in sterile ddH_2_O and they were used for the preparation of pesticide-supplemented MSM (10 mg L^-1^). In contrast, MSM supplemented with the more lipophilic carbaryl was prepared as described by Karpouzas et al., (2000). Triplicates containing MSM plus one of the compounds (10 ml) were inoculated with the degrading isolate. Triplicate non-inoculated controls were also prepared for each compound. Viable cell counts by serial dilution plating in NA gave an initial inoculum density of 2 × 10^6^ cfu ml^-1^. Degradation of pesticides was determined by regularly removing and analyzing samples by HPLC.

### Detection and Isolation of a Carbamate-Hydrolase Gene

Total DNA from the oxamyl-degrading bacterial isolates was screened for the presence of the known carbamate – hydrolase genes *mcd* and *cehA.* The primers used for this initial screening are listed in **Table 1**. Thermocycling conditions were as follows: Initial denaturation at 95°C, followed by 35 cycles of 30 s denaturation at 95°C, 30 s annealing at 58°C (*mcd*) or 53°C (*cehA*), 1 min extension at 72°C, followed by a final extension for 5 min at 72°C. The concentrations of the PCR reagents were: 200 μM of each dNTP (HT Biotechnology, UK), 1X polymerase buffer, 1.5 mM MgCl_2_, 1 U DNA polymerase (Finnzymes, Finland), 0.2 μM of each primer and sterile ddH_2_O.

**Table 1:** The sequences of the primers used for the detection and quantification of the carbamate hydrolase genes.

Gene	Primer	Sequence (5′–3′)	Purpose	Strains amplified	Fragment length (bp)	Reference
*cehA*	RTcehAf	ACCAACGCTCTACCAAATTACG	RT-q-PCR *cehA*	All	156	This study
	RTcehAr	GCAGTTGAGCAGATGATACCAC				This study
*gyrB*	RTgyrBf_P	CACCTGGTGGGTTTCCGTTC		OXA17, OXA18, OXA25	179	This study
	RTgyBr_P	CAGCTTGTCCTTGGTCTG				This study
	RTgyrBf_Pjin	CCTTCCACAACATTCATTTCAG		OXA20	169	This study
	RTgyrBr_Pjin	TGTTGGTGTTCAGGTATTCGAC				This study
*cehA*	cehAf	GATGATCCGTCACATAAG AGG	PCR detection	All	552	This study
	cehAr	GCAGTTGAGCAGAT GATACC				This study
*mcd*	mcdL1	CAAGAACTCAAATCCATCTACCTTGCC		All	561	Parekh et al. (1996)
	mcdL2	ATCCTTCCCTCGGAATGAATCGTCTCG				
*cehA*	cehAFf	TTGGACCAACCATTCAAACCAG	PCR amplification	All	2385	This study
	cehAFLr	TCACGTTAAGTCGCTTTCGGCGA	of full length gene			

Upon initial detection of the *cehA* gene in the DNA of the oxamyl-degrading isolates, the full length *cehA* gene (2385 bp) was obtained by PCR using primers cehAFLf – cehAFLr, which were designed based on the full length *cehA* gene sequence of *Rhizobium* sp. AC100 (Accession No. AB069723; **Table 1**). Thermocycling conditions were as follows: initial denaturation at 95°C, followed by 35 cycles of 30 s denaturation at 95°C, 30 s annealing at 55°C, 2.5 min extension at 72°C, followed by a final extension step for 10 min at 72°C. The concentrations of the reagents in the PCR reaction were as above with the only exception that 0.4 μM of each primer were utilized. The PCR products obtained were purified, cloned and transformed. Clones identified as positive were further processed for plasmid extraction and sequencing. The sequences of the *cehA* gene obtained were deposited in the EMBL database under the accession numbers FR751310 – FR751313.

### Transcription Analysis of the *cehA* Gene in Oxamyl-degrading Bacteria

The involvement of *cehA* gene in the hydrolysis of oxamyl was verified by following its expression during degradation of oxamyl. Thus, bacteria were inoculated in triplicate MSM + oxamyl (230 μM) and MSMN + succinate (0.1%). Triplicate non-inoculated controls were also included to determine the abiotic hydrolysis of oxamyl. Immediately prior to inoculation and at regular intervals thereafter, samples (1–5 ml) were removed from the flasks and used for the determination of oxamyl degradation and for RNA extraction. RNA was extracted with the Nucleospin RNA II kit (Macherey-Nagel, Düren, Germany) according to the manufacturers’ instructions. A DNAse treatment step (DNAse I, Amplification Grade, Invitrogen Life Technologies) was essential to remove DNA residues from extracted RNA. The absence of DNA contamination was further confirmed by PCR of the 16S rRNA gene. DNA-free RNA was then reverse-transcribed to obtain cDNA (kit Superscript II, Invitrogen Life Technologies) using random hexamers (Takara, Shiga, Japan).

The expression of the *cehA* gene was measured by RT-q-PCR using *gyrB* as a reference gene. New primers sets for the RT-q-PCR analysis of the *cehA* and *gyrB* genes were designed using the program Primer3 (**Table 1**). A single primer pair was designed for the amplification of the *cehA* gene from all oxamyl-degrading isolates, while two primer pairs were prepared to successfully amplify the *gyrB* gene of the different isolates. The specificity of the primers designed was verified by q-PCR using DNA from the targeted bacterial strains. Real-time PCR reaction mixtures contained 10 μl 2x SYBR Green PCR MasterMix (Kapa, Finland), 20 pmoles of each primer,1x ROX Low, 1 μl template cDNA and sterile distilled water to a total volume of 20 μl. Thermal conditions were 95°C for 3 min followed by 45 cycles of 95°C for 15 s and 63°C for 1 min. For detection of primer dimerisation or other artifacts of amplification, a melting-curve analysis was performed immediately after completion of the real-time PCR (95°C for 15 s, 55°C for 30 s, and then slowly increasing the temperature to 95°C). All reactions were performed in triplicate. Three non-template controls were included for each primer pair. Quantification of *cehA* gene expression was performed according to Pfaﬄ (2001).

### Utilization of Methylamine by Oxamyl-degrading Isolates

Considering that the hydrolysis of most carbamates results in the release of methylamine (Topp et al., 1993), the isolated oxamyl-degrading bacteria were tested for their ability to grow on methylamine. In addition, the oxamyl-degrading bacteria were screened for the presence of the *mauA* gene, which encodes methylamine dehydrogenase. This enzyme is commonly found in methylotrophic bacteria, which utilize methylamine as a C and N source (Chistoserdova, 2011). Thus, oxamyl-degrading bacteria were grown in MSM + oxamyl to mid-logarithmic phase. The bacterial pellet was harvested by centrifugation, washed three times with sterile ddH_2_O and used for the inoculation of duplicate MSM + oxamyl and MSM + methylamine (100 μg mL^-1^) agar plates. Growth on MSM without any C or N source was also tested to avoid false positives. The bacteria were sub-cultured at least three times in the corresponding media to ensure that their growth was purely on methylamine. The presence of the *mauA* gene in the oxamyl-degrading isolates was investigated via PCR using primers mauAII-232f (AAGTCTTGCGATTACTGGCG) and mauAII-526r (GACCGTGCAATGGTAGG TCA) (Hung et al., 2012). The *mauA* gene sequences obtained from the oxamyl-degrading isolates were deposited in the EMBL database under the accession numbers KT808462-KT808464.

### Pesticides Analysis

Extraction of oxamyl and its hydrolysis products from soil was performed as described by Karpouzas and Walker (2000b). For the determination of oxamyl and its derivatives in liquid cultures, aliquots of 0.8 ml were mixed with 0.2 ml of acetonitrile. The mixture was vortexed briefly and injected in an HPLC-UV system equipped with a GraceSmart RP C18 column (150 mm × 4.6 mm; Grace Davison Discovery Sciences, USA). Oxamyl and oxamyl oxime were detected at 220 nm using a mobile phase of acetonitrile:water (20:80 by volume) at flow rate of 1 ml min^-1^. Under these chromatographic conditions oxamyl and oxamyl oxime showed retention times of 3.1 and 2.3 min, respectively.

For the extraction of the other pesticides studied, aliquots of 0.5 ml of the liquid cultures were mixed with 0.5 ml of methanol. The mixture was vortexed briefly and it was directly analyzed by HPLC. For the elution of carbofuran and carbaryl, a mobile phase of acetonitrile:water (40:60 v:v) was used with UV detection at 215 nm. The retention times for carbofuran and carbaryl were 5.3 and 6.1 min, respectively. Methomyl was eluted using a mobile phase of acetonitrile:water (20:80 v:v) with UV detection at 235 nm and a retention time of 3.7 min. Aldicarb and its oxidation products (sulfoxide and sulfone) were analyzed as described by Karpouzas and Walker (2000b). More details regarding the quality controls of the analytical methods used are provided in the Supplementary Information.

## Results

### Degradation of Oxamyl and Oxamyl Oxime in Soil and Enrichment Cultures

**FIGURE 1:**
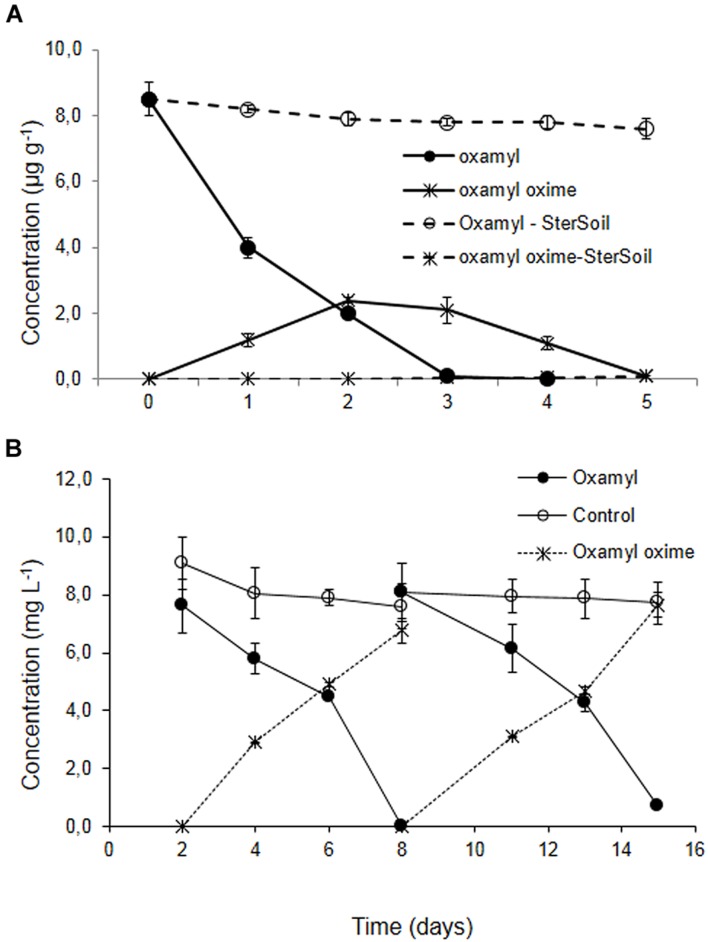
**(A)** The degradation of oxamyl and the formation of oxamyl-oxime in sterilized (dashed lines) and non-sterilized soil (solid lines) collected from a banana plantation in Crete with history of oxamyl treatments. **(B)** The degradation of oxamyl and the formation of oxamyl oxime in soil enrichment cultures in MSM and in non-inoculated controls. For clarity, only the degradation of oxamyl and the formation of oxamyl oxime in the second and third enrichment cycles are presented. Each value is the mean of three replicates + standard deviation.

Degradation of oxamyl in the soil from the banana plantation proceeded rapidly with the concurrent formation of oxamyl oxime. The latter did not accumulate and it was fully degraded by day 5 (**Figure 1A**). The degradation of oxamyl in soil followed first order kinetics with a DT50 of 1.6 days. Oxamyl degradation and oxamyl oxime formation was negligible in the sterilized soil (**Figure 1A**).

Subsequent enrichment cultures inoculated with the soil from the banana plantation showed a rapid degradation of oxamyl, which coincided with the formation of oxamyl oxime. In contrast to soil studies, oxamyl oxime was not further transformed and accumulated in the medium at the end of each enrichment cycle (**Figure 1B**). Negligible degradation of oxamyl was evident in the non-inoculated enrichment cultures.

Soil sterilization resulted in a complete halting of the degradation of oxamyl oxime, compared to 75% degradation which was observed in the non-sterilized soil (Supplementary Figure S1). This degradation pattern was not reflected in the corresponding enrichment cultures in MSM and MSMN, where no appreciable degradation of oxamyl oxime was observed (See Supplementary Figure S2). Repeated attempts to re-establish enrichment cultures for oxamyl oxime were not successful.

### Isolation and Phylogenetic Classification of the Oxamyl-degrading Isolates

**FIGURE 2:**
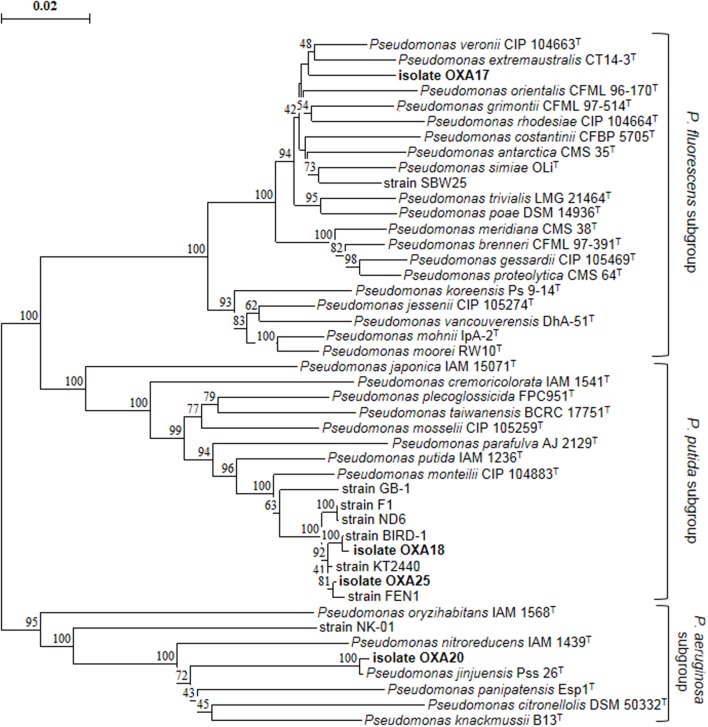
**Phylogenetic position of the isolated oxamyl-degrading isolates based on concatenated MLSA of the 16S rRNA, *gyrB*, and *rpoD* gene sequences.** Distance matrix was calculated by the Jukes and Cantor (1969) and the dendrogram was constructed by the neighbor-joining method (Saitou and Nei, 1987). Numbers on the nodes denote % bootstrap values based on 1,000 replicates. Scale bar represents 0.02 substitutions per site. *Pseudomonas* genus sub-groups were as defined by Mulet et al. (2010).

Enrichment cultures resulted in the isolation of four pure bacterial cultures which showed complete degradation of oxamyl within 7 days. MLSA based on partial sequences of the 16S rRNA, *gyrB*, and *rpoD* genes showed that all strains belonged to the genus *Pseudomonas* (**Figure 2**). In particular, strain OXA17 clustered within the *Pseudomonas fluorescens* subgroup and showed closest similarity to *P. extremaustralis* type strain. In contrast, strains OXA18 and OXA25 grouped within the subgroup of *P. putida* and showed closest similarity to *P. monteilii* type strain. Finally strain OXA20 clustered within the *P. aeruginosa* subgroup, and showed closest similarity to *P. jinjuensis* type strain. Based on these results the oxamyl-degrading bacteria were named as *P. extremaustralis* strain OXA17, *P. monteilii* strains OXA18 and OXA25, and *P. jinjuensis* strain OXA20.

### Growth Kinetics and Hydrolysis of Oxamyl

**FIGURE 3:**
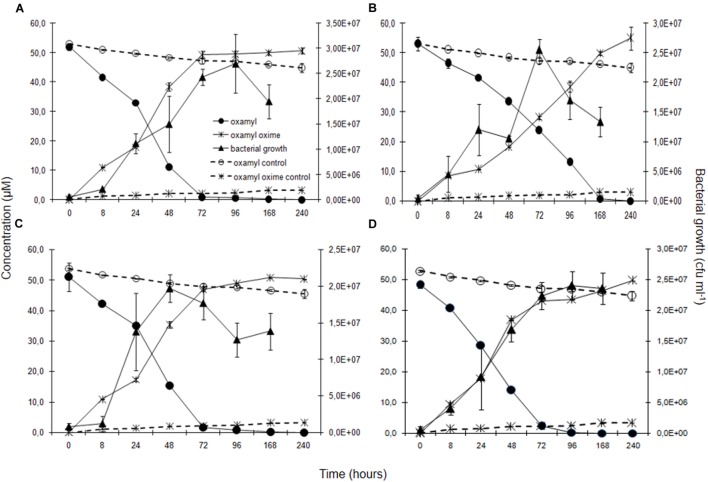
**The degradation of oxamyl and the formation of oxamyl oxime in MSM inoculated with strains OXA17 (A), OXA18 (B), OXA20 (C), and OXA25 (D).** The growth of the bacterial strains during degradation of oxamyl is also presented. Each value is the mean of three replicates with error bars showing the standard deviation of the mean.

Degradation of oxamyl was rapid and coincided with a build-up of bacterial growth, which reached to levels higher than 10^7^ cells ml^-1^ at 24 h in the cultures of all the oxamyl-degrading bacteria (**Figure 3**). Degradation of oxamyl by the strains OXA17, OXA20, and OXA25 was completed within 96 h (**Figures 3A,C,D**), while 7 days were needed for the degradation oxamyl by the strain OXA18 (**Figure 3B**). Oxamyl degradation coincided with the formation of oxamyl oxime which was accumulated in the liquid cultures. Extending the incubation to 21 days did not result in appreciable degradation of oxamyl oxime. Negligible hydrolysis of oxamyl to oxamyl oxime was observed in the non-inoculated control cultures (**Figure 3**).

### Degradation of Other Pesticides by the Oxamyl-degrading Strain OXA20

Strain OXA20 was able to completely degrade the oximino carbamates oxamyl and aldicarb, and the aryl-methyl carbamate carbaryl within 4 days, while a period of 7 days was required for the complete degradation of methomyl and carbofuran (**Table 2**). Negligible degradation of all tested pesticides (<10%) was observed in the non-inoculated controls (**Table 2**).

**Table 2:** The degradation (%) of the oximino carbamates oxamyl, aldicarb, and methomyl and of the aryl-methyl carbamates carbaryl and carbofuran in MSMN inoculated with the strain OXA20.

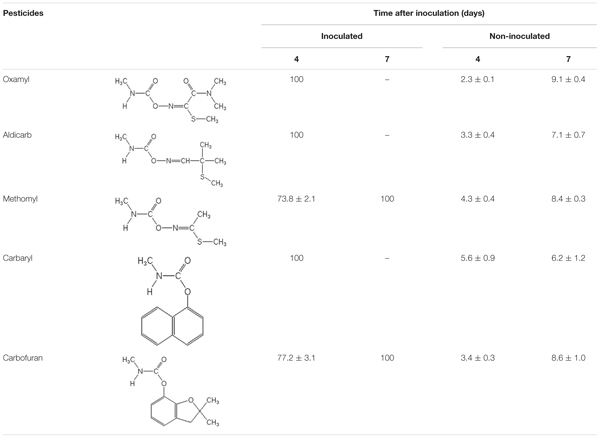

*The degradation of the pesticides in non-inoculated cultures is also presented. Each value is the mean of three replicates ± standard deviation*.

### Detection of a Carbamate-Hydrolase Gene in the Oxamyl-degrading Bacteria

**FIGURE 4:**
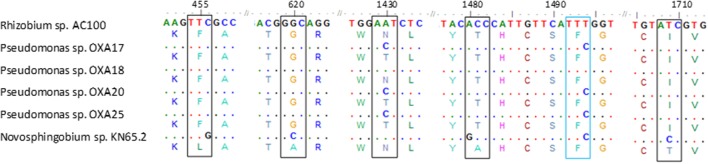
**Nucleotide (and amino acid) sequence alignment of the *cehA* genes of the oxamyl-degrading strains OXA17, OXA18, OXA20, and OX25, the carbaryl-degrading strain *Rhizobium* AC100 (Hashimoto et al., 2002; Accession No. AB069723) and the carbofuran-degrading strain *Novosphingobium* KN65.2 (Nguyen et al., 2014; Accession No. CCBH010000016, CDO34164.1).** Only the parts of the alignment where sequence divergence was observed are presented. Polymorphisms in the sequences of the *cehA* gene of the oxamyl-degrading strains OXA17, OXA18, OXA20, OXA25 and of the *Novosphingobium* strain KN65.2 compared to the sequence of the *cehA* gene of the *Rhizobium* strain AC100 are shown where blue color indicates sequence polymorphisms which do not translate to amino acid sequence polymorphisms, while black color indicates sequence polymorphisms which translate into respective amino acid polymorphisms. Dots represent nucleotide homology between the *cehA* genes compared.

PCR screening of total DNA from the oxamyl-degrading isolates for carbamate hydrolase genes gave positive amplification only for the *cehA* gene (Supplementary Figure S3). PCR amplification and sequencing of the full length *cehA* gene showed that all oxamyl-degrading isolates carried similar *cehA* genes. Further alignment of the *cehA* genes carried by the oxamyl-degrading isolates with the *cehA* gene sequences of the carbaryl-degrading strain *Rhizobium* AC100 and of the carbofuran-degrading strain *Novosphingobium* KN65.2 showed high levels of homology (**Figure 4**). In particular, the *cehA* gene of the strain OXA18 was identical to the *cehA* gene sequence of the *Rhizobium* strain AC100, while the *cehA* genes carried by the other three isolates were identical and differed only in two nucleotide positions (1430 and 1494) with the *cehA* gene of the *Rhizobium* strain AC100. However, only the nucleotide polymorphism at position 1430 resulted in a change in the amino acid sequence of the resulting protein (threonine instead of asparagine). In contrast, the *cehA* gene sequence of *Novosphingobium* sp. KN65.2 was more diverse and showed four nucleotide difference with the *cehA* sequence of the strains OXA17, OXA20, and OXA25, and five nucleotide difference with the *cehA* sequences of the strain OXA18 and *Rhizobium* AC100 (**Figure 4**).

### Transcription Analysis of the *cehA* Gene in Oxamyl-degrading Bacteria

**FIGURE 5:**
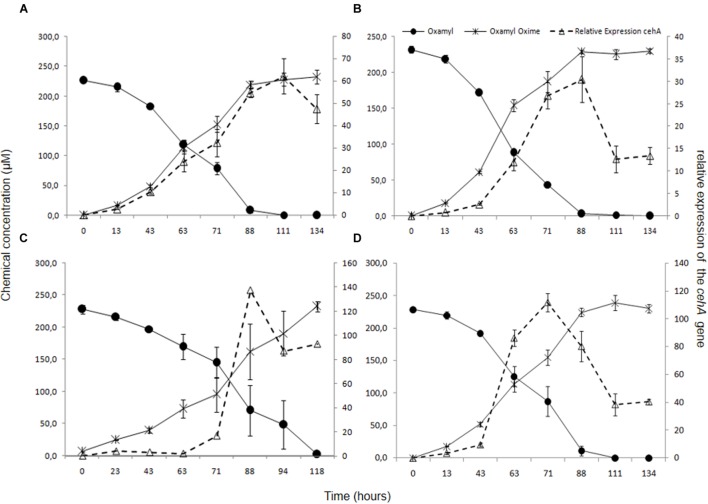
**The hydrolysis of oxamyl to oxamyl oxime and the relative expression of the *cehA* gene in MSM inoculated with the strains OXA17 (A), OXA18 (B), OXA 20 (C), and OXA25 (D).** The relative expression of the *cehA* gene was calculated as a ratio of its expression in MSM + oxamyl to its expression in MSMN + succinate. Each value is the mean of three replicates with error bars representing the standard deviation of the mean.

In order to verify the functional role of the *cehA* gene in the degradation of oxamyl, its transcription during degradation of oxamyl was followed via RT-q-PCR. The relative expression of the *cehA* gene (ratio of its expression in MSM + oxamyl to its expression in MSMN + succinate) followed the same temporal pattern in all oxamyl-degrading strains (**Figure 5**). The relative expression of *cehA* increased concurrently with the hydrolysis of oxamyl and reached to maximum levels at 71 h (OXA25), 88 h (OXA17, OXA20), or 111 h (OXA 17). For instance, the expression of the *cehA* gene in the strains OXA20 and OXA25 was 140- and 120-times higher in the oxamyl-amended cultures compared to the corresponding succinate-amended cultures (**Figures 5C,D**).

### Utilization of Methylamine by Oxamyl-degrading Bacteria

All isolates were able to grow on MSM + methylamine upon numerous transfers. PCR tests showed positive amplification of the *mauA* gene in three of the four oxamyl-degrading strains (OXA18, OXA20, and OXA25; Supplementary Figure S4). Sequencing of the PCR products obtained showed highest sequence match to the methylamine dehydrogenase of *Methylobacterium extorquens* strain AM1 (91% at the DNA level, Accession no. CP001510). Further attempts to amplify the *mauA* gene from the DNA of the strain OXA17 using alternatively primers (Neufeld et al., 2007) and optimization of the PCR conditions did not result in a successful amplification.

## Discussion

A rapid microbially driven hydrolysis of oxamyl was observed in a soil with history of previous treatment with oxamyl. This is in line with the reduced biological efficacy of oxamyl in the given field, as reported by the grower. Previous studies have demonstrated the vulnerability of oxamyl to enhanced microbial degradation, which could lead to unacceptable reduction in its biological efficacy (Smelt et al., 1987, 1996).

Enrichment cultures from the adapted soil resulted in the isolation of four bacterial strains, which were all identified, based on MLSA, as *Pseudomonas*. Earlier studies have reported the isolation of pseudomonads rapidly degrading a range of pesticides including organophosphates (Karpouzas et al., 2000), carbamates (Bano and Musarrat, 2006), and pyrethroids (Mallic et al., 2009). Our study constitutes only the second report regarding the isolation of oxamyl-degrading bacteria. Osborn et al. (2010b) isolated several oxamyl-degrading strains from soils exhibiting enhanced biodegradation of oxamyl. Their isolates were phylogenetically assigned to the genera *Aminobacter* and *Mesorhizobium*. However, these authors did not provide any information regarding the metabolic pathway of oxamyl. Our isolates were able to metabolize oxamyl via hydrolysis of its methyl-carbamoyl moiety to form oxamyl oxime, which was not further transformed neither in the soil enrichment cultures nor by the isolated bacteria. Previous metabolism studies in soil have also identified oxamyl oxime as the main metabolite of oxamyl (Ou and Rao, 1986); however, this is the first report for the formation of oxamyl oxime by soil bacteria. The accumulation of oxamyl oxime in enrichment and pure cultures is in contrast to its gradual dissipation in soil (**Figure 1A**). Sterilization of soil halted the degradation of oxamyl oxime suggesting that its transformation was biologically driven. Attempts to isolate oxamyl oxime – degrading bacteria following the same enrichment culture method failed, suggesting that its transformation in soil is probably a co-metabolic process performed by non-specialized soil bacteria or fungi. Either way, the hydrolysis of oxamyl is considered a detoxification step since oxamyl oxime is more than an order of magnitude less toxic to mammals, aquatic organisms and earthworms compared to the parent compound (European Food Safety Authority [EFSA], 2005).

**FIGURE 6:**
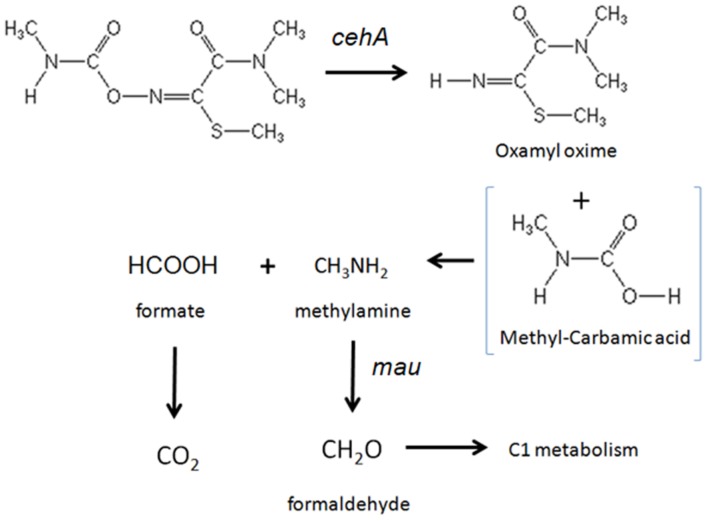
**The proposed metabolic pathway of oxamyl by the isolated bacteria.** The steps of the metabolic pathway controlled by the genes *cehA* and methylamine dehydrogenase (*mau*) are indicated.

The accumulation of oxamyl oxime in the bacterial cultures indicates that the oxamyl-degrading isolates were not able to exploit this metabolic product as an energy source. Thus it seems probable that the oxamyl-degrading isolates could utilize the methyl-carbamoyl moiety that is released during hydrolysis of oxamyl as a C and N source. This pathway is common among carbamate-hydrolyzing bacteria (Karns et al., 1986; Chaudhry and Ali, 1988; Hashimoto et al., 2006) and leads to the transient formation of carbamic acid, which is unstable and it is rapidly broken down to methylamine and CO_2_ (Karns et al., 1986; Feng et al., 1997; Bachman and Patterson, 1999). The former product could be utilized as a C and N source through the C1 metabolism of bacteria (Chistoserdova et al., 2009). This is in line with the proven ability of our isolates to utilize oxamyl as C and N source and with their capacity to grow on methylamine. Methylotrophy has been identified as a common feature of carbofuran-degrading bacteria (Chaudhry and Ali, 1988; Singh et al., 1993; Topp et al., 1993). Trabue et al. (2001) demonstrated that the use of 2 μg g^-1^ of carbofuran in an adapted soil could lead to the production of adequate amount of methylamine to sustain a significant increase in the carbofuran-degrading microbiota using it as a C and N source. Further evidence for the capacity of our isolates to utilize methylamine was provided by the detection of a methylamine dehydrogenase gene in three of the four oxamyl-degrading strains. Methylamine dehydrogenase is one of the hallmark genes in methylotrophy responsible for the oxidation of methylamine to formaldehyde, which is then assimilated via different paths (Chistoserdova, 2011). The non-detection of methylamine dehydrogenase in the strain OXA17 might be a result of the limited coverage of the primers utilized or the presence of an alternative pathway, like the *N*-methylglutamate oxidation pathway of methylamine, which has been found in many proteobacteria (Latypova et al., 2010). Based on all the above a microbial metabolic pathway of oxamyl is proposed (**Figure 6**).

The oxamyl-degrading strains were found to possess the carbamate-hydrolase gene *cehA*, which was first detected in a plasmid of the carbaryl-degrading *Rhizobium* strain AC100 (Hashimoto et al., 2002). Recently the *cehA* gene was detected in the carbofuran-degrading *Novosphingobium* strain KN65.2, which constitutes the only carbamate-degrading bacterium whose genome sequence is available (Nguyen et al., 2014). The *cehA* gene sequences of the oxamyl-degrading strains were identical (OXA18) or nearly identical (OXA17, OXA20, and OXA25) to the *cehA* gene sequence of the *Rhizobium* strain AC100 (Hashimoto et al., 2002). Whereas a higher sequence divergence was observed with the *cehA* gene sequence of the carbofuran-degrading *Novosphingobium* strain KN65.2 (Nguyen et al., 2014). However, the latter study did not provide evidence for the involvement of this gene in the degradation of carbofuran, in contrast to our study where transcription analysis demonstrated its direct involvement in the hydrolysis of oxamyl. The environmental relevance of *cehA* for the soil biodegradation of oxamyl is supported by recent studies which observed a significant positive correlation between the degradation rates of oxamyl in 16 agricultural soils from a potato monoculture area in Crete and the abundance of the *cehA* gene (Rousidou et al., 2014).

Strain OXA20, selected as a representative of the oxamyl-degrading isolates available, rapidly degraded both the oximino carbamates aldicarb and methomyl, structurally similar to oxamyl, and the aryl-methyl carbamates carbofuran and carbaryl, which resemble oxamyl only at the methyl-carbamoyl moiety. These results further support our initial suggestion that these bacteria utilize the methyl-carbamoyl moiety produced by the hydrolysis of oxamyl as C and N source. The relaxed substrate specificity exhibited by the strain OXA20 is not common among carbamate-degrading bacteria. Yan et al. (2007) isolated a carbofuran-degrading *Novosphingobium* strain, which was able to degrade carbaryl but not methomyl. Similarly, a methylotrophic carbofuran-degrading strain was able to metabolize carbaryl but not methomyl and aldicarb (Topp et al., 1993). Although the protein encoded by the *cehA* gene of our isolates was not obtained and tested, the capacity of the strain OXA20 to degrade carbofuran and other carbamates is against the lack of carbofuran degradation activity by the *Rhizobium* strain AC100 (its capacity to degrade oximino carbamates like oxamyl was not tested; Hashimoto et al., 2002). The single amino acid difference in the sequence of the CEHA of the *Pseudomonas* strain OXA20 (OXA17 and OXA25) compared to the CEHA of the *Rhizobium* strain AC100 might be responsible for the different substrate specificity observed. This remains to be verified by isolation and heterologous expression of the hydrolase encoded by the *cehA* gene.

## Conclusion

We report the isolation and identification of four oxamyl-degrading *Pseudomonas* strains. The isolated bacteria were capable of hydrolyzing oxamyl to oxamyl oxime, which was not further transformed, and methylamine which was utilized as a C and N source. All the strains isolated carried the *cehA* gene, a carbaryl hydrolase gene, which was shown by transcription analysis to be responsible for the hydrolysis of oxamyl.

## Author Contributions

KR and EC performed most of the laboratory work and KR contributed on the writing of the paper. DG, ES, DK, and PK contributed parts of the laboratory work. SN and MT performed the phylogenetic analysis of the isolated bacteria and the isolated hydrolase genes. ET identified and collected the soils suffering from enhanced biodegradation from where the bacteria where isolated. DK formed the idea and supervised the work.

## Conflict of Interest Statement

The authors declare that the research was conducted in the absence of any commercial or financial relationships that could be construed as a potential conflict of interest.

## Footnotes

1 http://www.ebi.ac.uk/Tools/msa/clustalo/
