# Supplementary material for: Isolation of Oxamyl-degrading Bacteria and Identification of cehA as a Novel Oxamyl Hydrolase Gene
Source: Front Microbiol. 2016 Apr 29;7:616. doi: 10.3389/fmicb.2016.00616 (PMC4850150; doi:10.3389/fmicb.2016.00616)
Supplement: Supplementary file 1 [file Table_1.DOCX]

**Supplementary Table S1.** The primers used for the amplification of the genes utilized for phylogenetic analysis of the bacterial isolates

| **Gene** | **Primer** | **Sequence (5’- 3’)** | **Strains amplified** | **Fragment length (bp)** | **Reference** |
| --- | --- | --- | --- | --- | --- |
| 16S rRNA | 8f | AGA GTT TGA TCC TGG CTC AG | All | 1502 | Felske et al. 1997 |
|  | 1512r | ACGGCTACCTTGTTACGACTT |  |  | Felske et al. 1997 |
| *gyrB* | UP1E | AYGSNGGNGGNARTTYRA | OXA17, OXA18, OXA25 | 966 | Yamamoto *et al.*, 2000 |
|  | APrU | GCNGGRTCYTTYTCYTGRCA |  |  | Yamamoto *et al.*, 2000 |
|  | gBMM1F | GTGTCGGTKGTRAACGCCC | OXA20 |  | Mulet et al. 2010 |
|  | gBMM725R | GCYTCRTTSGGRTTYTCCAGCAGG |  |  | Mulet et al.2010 |
| *rpoD* | PsEG30F | ATYGAAATCGCCAARCG | All | 760 | Mulet et al, 2009 |
|  | PsEG790R | CGGTTGATKTCCTTGA |  |  | Mulet et al, 2009 |
