# Supplementary material for: Isolation of Oxamyl-degrading Bacteria and Identification of cehA as a Novel Oxamyl Hydrolase Gene
Source: Front Microbiol. 2016 Apr 29;7:616. doi: 10.3389/fmicb.2016.00616 (PMC4850150; doi:10.3389/fmicb.2016.00616)
Supplement: Supplementary file 2 [file Presentation_1.PPTX]

## Slide 1
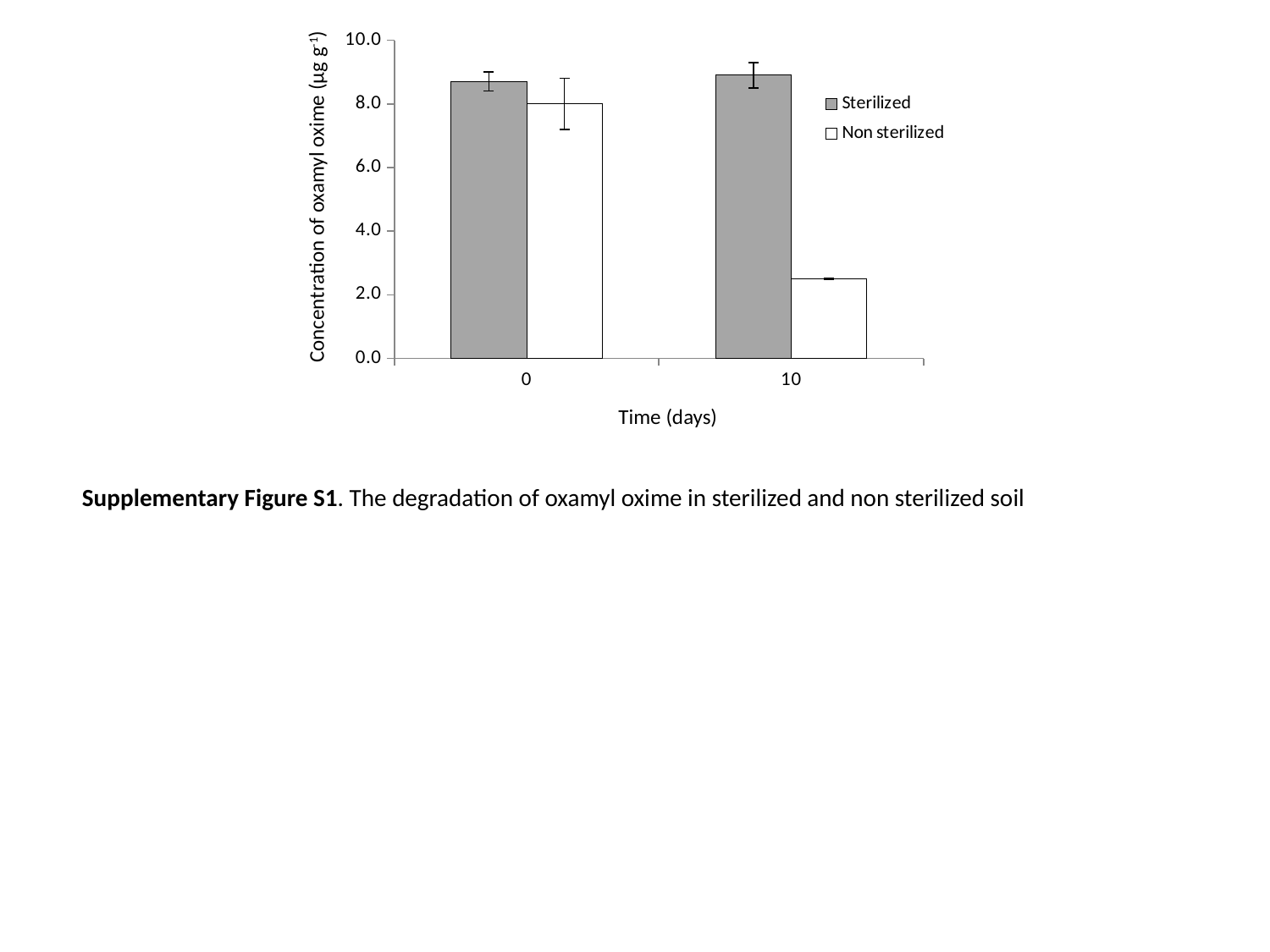

### Chart
| Category | Sterilized | Non sterilized |
|---|---|---|
| 0 | 8.700000000000001 | 8.0 |
| 10 | 8.9 | 2.5 |Concentration of oxamyl oxime (μg g-1)
Time (days)
Supplementary Figure S1. The degradation of oxamyl oxime in sterilized and non sterilized soil

## Slide 2
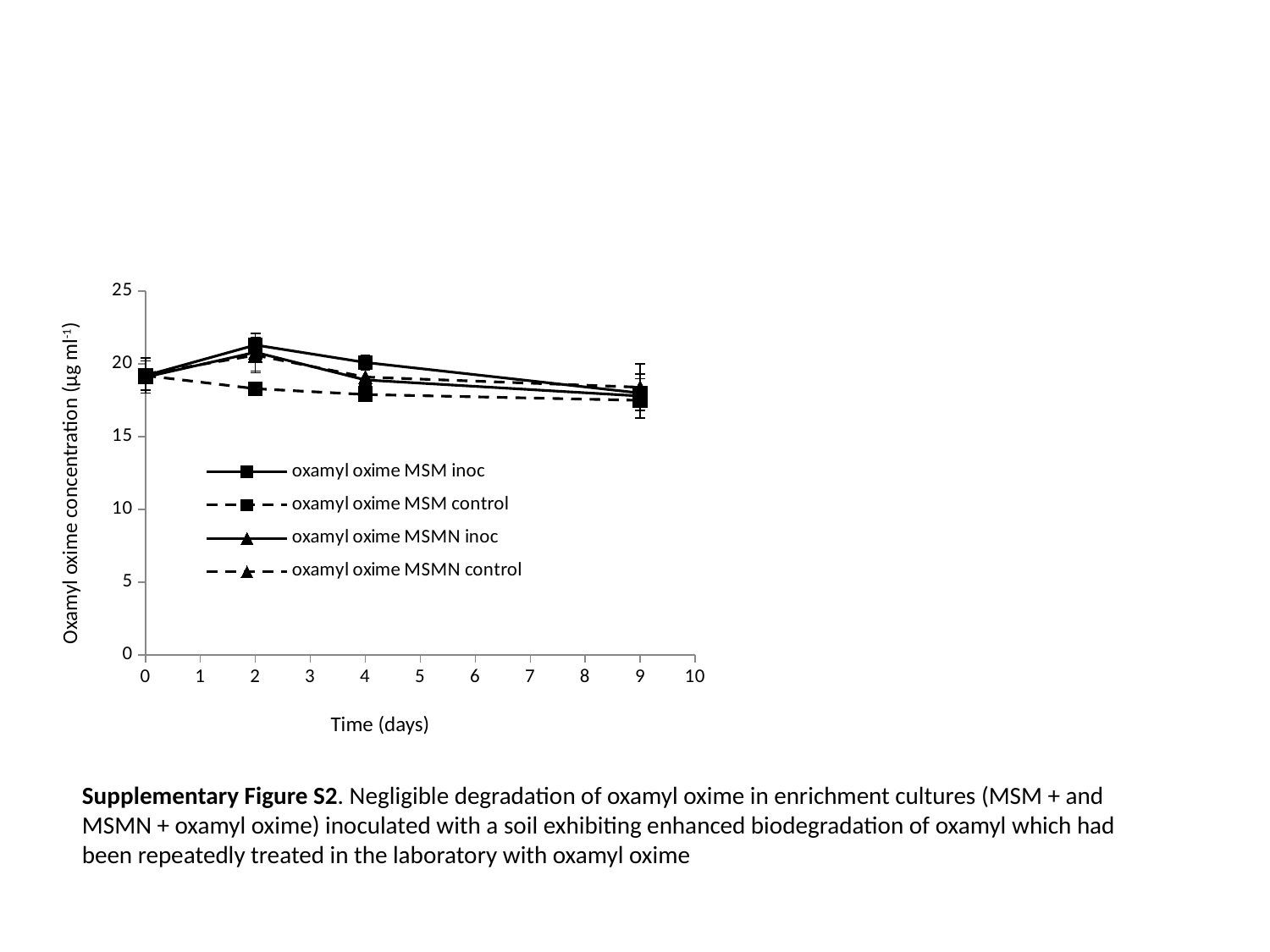

### Chart
| Category | oxamyl oxime MSM inoc | oxamyl oxime MSM control | oxamyl oxime MSMN inoc | oxamyl oxime MSMN control |
|---|---|---|---|---|Oxamyl oxime concentration (μg ml-1)
Time (days)
Supplementary Figure S2. Negligible degradation of oxamyl oxime in enrichment cultures (MSM + and MSMN + oxamyl oxime) inoculated with a soil exhibiting enhanced biodegradation of oxamyl which had been repeatedly treated in the laboratory with oxamyl oxime

## Slide 3
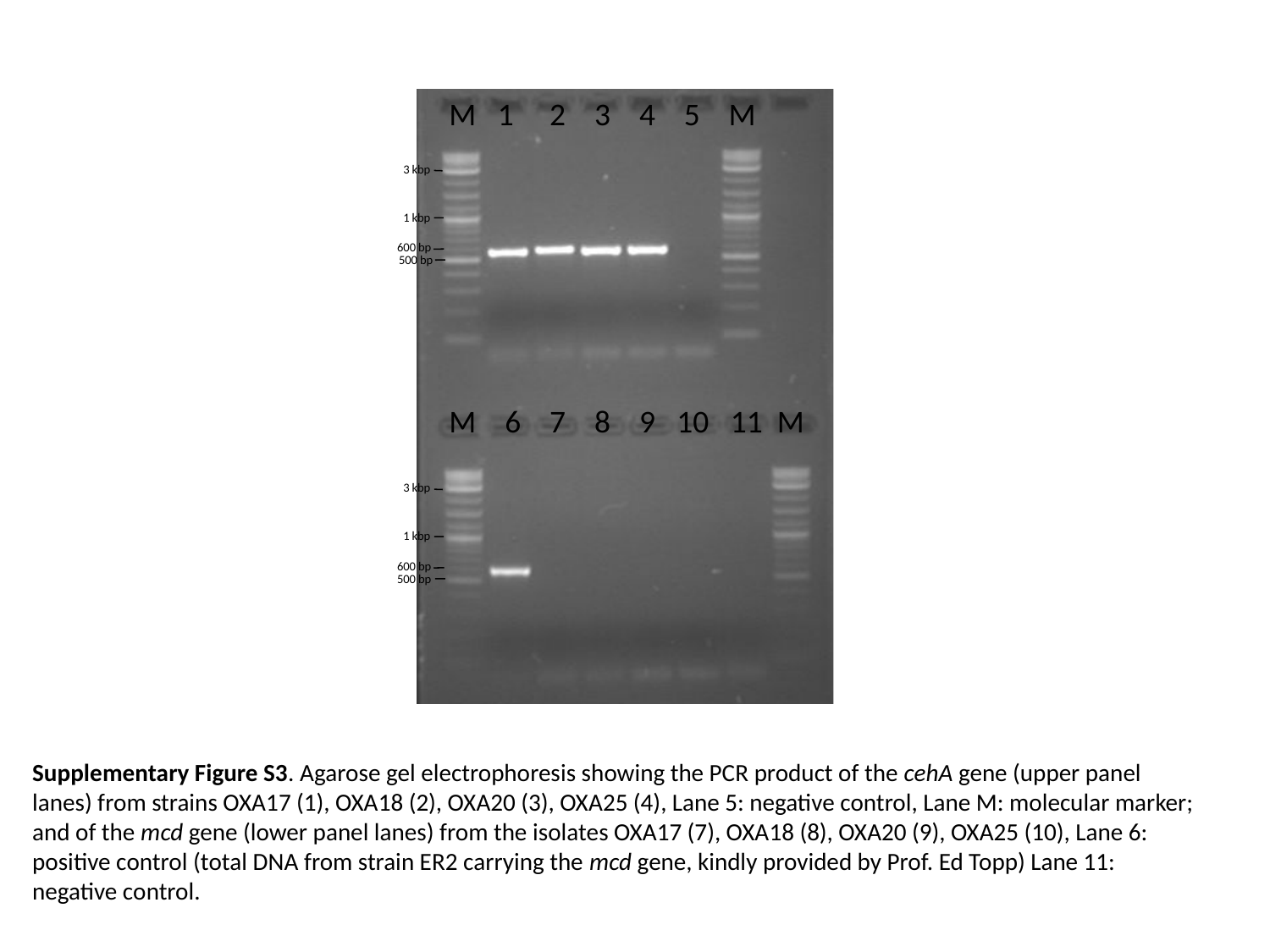

M 1 2 3 4 5 M
3 kbp
1 kbp
600 bp
500 bp
M 6 7 8 9 10 11 M
3 kbp
1 kbp
600 bp
500 bp
Supplementary Figure S3. Agarose gel electrophoresis showing the PCR product of the cehA gene (upper panel lanes) from strains OXA17 (1), OXA18 (2), OXA20 (3), OXA25 (4), Lane 5: negative control, Lane M: molecular marker; and of the mcd gene (lower panel lanes) from the isolates OXA17 (7), OXA18 (8), OXA20 (9), OXA25 (10), Lane 6: positive control (total DNA from strain ER2 carrying the mcd gene, kindly provided by Prof. Ed Topp) Lane 11: negative control.

## Slide 4
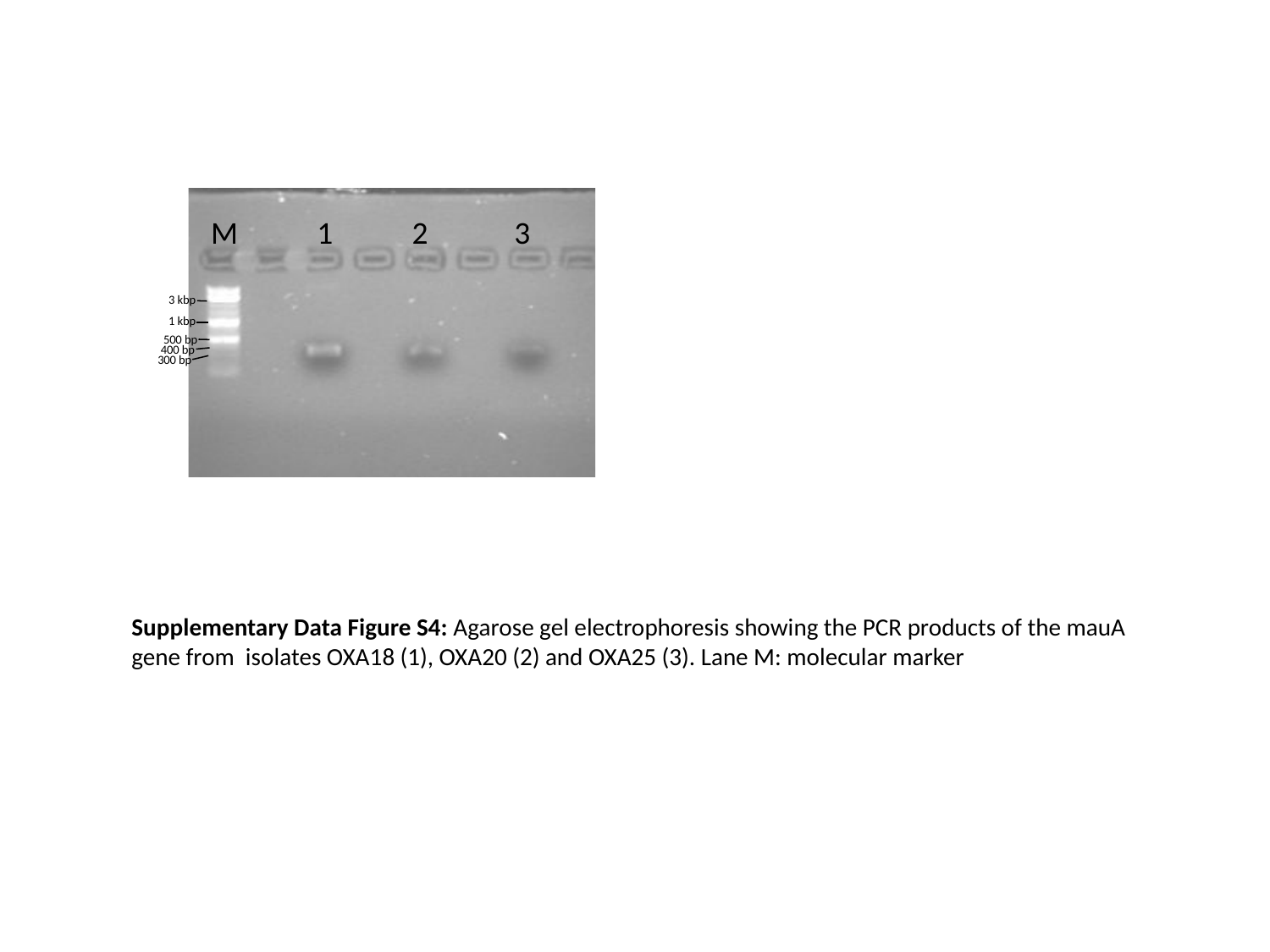

M 1 2 3
 3 kbp
 1 kbp
500 bp
400 bp
300 bp
Supplementary Data Figure S4: Agarose gel electrophoresis showing the PCR products of the mauA gene from isolates OXA18 (1), OXA20 (2) and OXA25 (3). Lane M: molecular marker
