# Supplementary material for: Isolation of Oxamyl-degrading Bacteria and Identification of cehA as a Novel Oxamyl Hydrolase Gene
Source: Front Microbiol. 2016 Apr 29;7:616. doi: 10.3389/fmicb.2016.00616 (PMC4850150; doi:10.3389/fmicb.2016.00616)
Supplement: Supplementary file 3 [file Data_Sheet_1.DOCX]

**Supplementary information**

1. Materials and Methods

1.1. Pesticide Analysis

Oxamyl residues were extracted from soil (10 g) with methanol (20 ml) via agitation for an 1 h in an orbital shaker at 200 rpm. Samples were then centrifuged for 15 min at 4000 rpm for and the supernatant was collected, filtered through syringe filters and used for HPLC analysis as is described in the manuscript. Recovery tests at three fortification levels (0.05, 1 and 10 μg g^-1^) showed recoveries above 90% in all cases. The limit of quantification for oxamyl was 0.02 μg g^-1^. Regarding the extraction of oxamyl and of the other pesticides tested from liquid cultures, recovery tests at three fortification levels (0.05, 5 and 20 μg ml^-1^) showed recoveries of 85-98%, 87-92%, 83-89%, 95-101%, 82-91% and 81-99% for oxamyl, carbofuran, carbaryl, methomyl, aldicarb and fenamiphos respectively. The limit of detection for the different pesticides tested was 0.02 μg ml^-1^.

Quantification of pesticide residues was performed by the external standard method using the calibration curves obtained by the injection of standard solutions of the pesticides studied. Good linearity responses were obtained for all the compounds in the ranges studied (0.02–10 μg ml^−1^).
